# Computational model for drug research

**DOI:** 10.1093/bib/bbae158

**Published:** 2024-04-05

**Authors:** Xing Chen, Li Huang

**Affiliations:** School of Science, Jiangnan University, Wuxi, 214122, China; The Future Laboratory, Tsinghua University, Beijing, 100084, China

**Keywords:** drug research, computational model, interaction prediction, drug treatment, drug interaction

## Abstract

This special issue focuses on computational model for drug research regarding drug bioactivity prediction, drug-related interaction prediction, modelling for immunotherapy and modelling for treatment of a specific disease, as conveyed by the following six research and four review articles. Notably, these 10 papers described a wide variety of in-depth drug research from the computational perspective and may represent a snapshot of the wide research landscape.

Driven by accumulation of biochemical data [1–3] as well as advances in deep learning and graphics processing units (GPUs) [4, 5], modern computer-aided drug design exhibits considerably improved efficacy [6–10] in more diversified applications [11–13], with increasing success stories of clinical-phase compounds or approved drugs [14–16]. This special issue focuses on computational model for drug research regarding bioactivity prediction, drug-related interaction prediction, modelling for immunotherapy and modelling for treatment of a specific disease, as conveyed by the following six research and four review articles. Notably, these 10 papers may represent a snapshot of the wide research landscape.

Yin *et al.* [17] put forward a novel algorithm named adversarial feature subspace enhancement (AFSE) that complemented deep graph learning (DGL) models for predicting ligand bioactivities (targeting GPCR proteins) to improve their generalization in real virtual screening scenarios. When applied to DGL models, AFSE deployed bi-directional adversarial learning to dynamically produce plentiful subspace representations, followed by minimizing the maximum loss of molecular divergence and bioactivity to guarantee local smoothness of model outputs, hence promoting generalization.

Zhang *et al.* [18] concentrated on predicting bioactivities of drug inactive ingredients (DIGs) in drug formulations (DFMs). Since such bioactivity data were not ready-made, they firstly curated vast DIGs and DFMs from public resources and literature into a novel database named biological ACtivities of Drug INActive ingredients (ACDINA), where the entries’ targets were cross-linked to other databases containing their pharmaceutical/biological characteristics. Then, ACDINA was utilized to construct five classical machine learning and two deep learning models for predicting DIGs’ bioactivities and evaluating the viability of adopting the proposed database on drug discovery and precision medicine.

Wang *et al.* [19] comprehensively overviewed the recent advances in data resources and computational models for inferring correlations between microbes, drugs and diseases. Specifically, major databases related to microbe–disease associations, drug–disease associations and microbe–drug associations were introduced before systematically presenting five categories of models, including network, matrix factorization, matrix completion, regularization and artificial neural network. The review concluded with an outline of research challenges, opportunities and suggestions for future works.

Feng *et al.* [20] designed a model named Directed Graph Attention Network for predicting asymmetric Drug–Drug Interactions that employed an encoder to learn asymmetric embeddings of the source, target and self-roles of a drug, calculated aggressiveness/impressionability (role-specific items) to capture interaction tendency of the source/target role and used a predictor to identify direction-specific interactions between two drugs by combining the embeddings and role-specific items.

Luo et *al.* [21] performed large-scale literature mining on PubMed to retrieve MeSH diseases, MeSH drugs and Entrez genes that formed the data basis for building a Drug Set Enrichment Analysis by Text Mining (DSEATM) pipeline. It obtained each investigated disease’s associated drugs and their target genes and utilized the latter to enrich the pathways for the disease. DSEATM was evaluated against several state-of-the-art methods to confirm its effectiveness and potential in disease and drug research.

Rintala *et al.* [22] provided an overview of network methods for modelling the effect of drugs and diseases to reveal the formers’ mechanism of action and therapeutic effects. Descriptions of methods for constructing different useful biological networks preceded the introduction of network data mining (NDM) algorithms, including graph search, centrality, path finding, community detection, machine learning and graph embeddings. Then, applications of NDM to drug discovery were elucidated with a detailed discussion on drug repurposing for COVID-19.

Li *et al.* [23] developed HLA3D, an integrated toolkit for the three-dimensional structure analysis of human leukocyte antigen (HLA) molecules to enhance the HLA data application in transplantation and tumour immunotherapy. The toolkit consisted of a risk alignment pipeline that offered alignment and visualization functions as well as risk reports of mismatched HLA molecules in organ transplantation and an antigenic peptide prediction pipeline that implemented mutation prediction, peptide prediction, immunogenicity assessment and docking simulation for tumour vaccine. Both pipelines integrated diverse bioinformatics resources and tools.

Wilman *et al.* [24] contributed a critical review on opportunities, feasibility and advantages of deep learning models used to assist in the therapeutic design of antibodies. Applications of such models to antibody structure prediction, antibody–antigen binding prediction and humanization of animal antibodies were outlined and analysed along with prospects on language-motivated antibody embeddings and automated antibody sequence generation.

Scafuri *et al.* [25] reviewed various computational models conducive to the search for pharmacological chaperones (PCs), a type of protein-stabilizing compounds for treating rare genetic diseases resulted from destabilizing mutations. The elaborated model categories include prediction of mutation impacts on protein stability, identification of alternative ligand-binding sites for non-competitive PC and virtual screening of potential PCs for target proteins.

Toro-Domínguez *et al.* [26] sought to combat the rare disease Systemic Lupus Erythematosus (SLE) and established a scoring system to measure the personalized Molecular dYregulated PROfiles of SLE patients. Representing patients’ transcriptome as immunological gene modules, the system computed a dysregulation score for each gene module according to averaged *z*-scores. Experiments involved 6100 SLE and 750 healthy samples for analysing correlations among dysregulation scores, clinical events and response to the Tabalumab antibody; based on the results, machine learning models were fitted to predict nearly 100 clinical parameters that might collectively achieve personalized medicine.

Apparently, the 10 papers in this special issue described a wide variety of in-depth drug research from the computational perspective. We express our gratitude to the authors for their contributions, the editorial staff members for their support and hard work and the anonymous reviewers for their valuable comments.

## Author Biographies

**Xing Chen**, PhD, is a professor of School of Science, Jiangnan University. His research interests include complex disease-related non-coding ribonucleic acid biomarker prediction, computational models for drug discovery and early detection of human complex disease based on big data and artificial intelligence algorithms.

**Li Huang** is a PhD student of The Future Laboratory, Tsinghua University. His research interests include bioinformatics, complex network algorithm, machine learning and visual analytics.
